# Reversible Focal Splenial Lesion on Diffusion–Weighted MRI in Sulfonylurea Intoxication

**DOI:** 10.5334/jbr-btr.826

**Published:** 2015-09-15

**Authors:** K. Aslan, A. V. Polat, G. O. Taskin, L. Incesu, R. Aydin

**Affiliations:** 1 Department of Radiology, University of Ondokuz Mayis, Faculty of Medicine, Samsun, Turkey; 2 Department of Radiology, Medicana Samsun Hospital, Samsun, Turkey; 3 Department of Radiology, Samsun Education and Research Hospital, Samsun, Turkey

**Keywords:** Poisons, poisoning

## Abstract

Hypoglycemic brain injury is usually reversible, and partial recovery or mortality depends on the affected area. Diffusion-weighted imaging (DWI) may be useful in predicting the prognosis according to the site of involvement. Isolated lesions of the splenium of corpus callosum (SCC) in hypoglycemic brain injury are very rare, and DWI findings of a reversible lesion of the SCC due to deep hypoglycemia associated with sulfonylurea intoxication has been reported only once in the literature. We report the case of a 15-year-old girl admitted to the emergency department who had attempted suicide using sulfonylurea and subsequently went into a coma. The patient had no known previous disease. Except for a blood glucose level of 10 mg/dl, all other blood laboratory tests were normal. DWI performed two hours after admission showed diffusion restriction in the SCC. After receiving treatment for 24 hours, the patient became conscious, and her blood glucose level returned to normal. Two days later, complete resolution of the SCC lesion was revealed by control DWI. We discuss both the DWI findings of the reversible SCC lesion due to hypoglycemic brain injury resulting from sulfonylurea intoxication and the role of DWI in predicting the clinical outcome.

Neurological findings of hypoglycemia vary from reversible focal deficits to permanent dysfunction or death [1,2,3]. According to MRI findings, the cerebral cortex, basal ganglion, hippocampus, internal capsule, splenium of corpus callosum (SCC), and cerebral white matter are the most commonly affected sites in hypoglycemic brain injury [2,3,4].

Hypoglycemic brain injury is usually reversible, and partial recovery or mortality depends on the affected area [2]. Several studies have shown that diffusion-weighted imaging (DWI) may be useful in predicting the prognosis according to the site of involvement [1,2,3,4]. Isolated lesion of the SCC in hypoglycemic brain injury are very rare in the literature, but those reported were found to be reversible, both clinically and radiologically [3,5]. To our knowledge, DWI findings of a reversible lesion of the SCC due to deep hypoglycemia associated with sulfonylurea intoxication has been reported only once in the literature [6]. We present the DWI findings of a reversible SCC lesion due to hypoglycemic brain injury resulting from sulfonylurea intoxication. We also discuss the possible pathogenesis of the lesion and the benefit of using DWI in predicting the clinical outcome.

## Case report

A 15-year-old girl who attempted suicide using sulfonylurea, an oral hypoglycemic drug and subsequently became comatose was admitted to the pediatric emergency department. The patient had no known previous disease. On admission, vital signs (blood pressure, pulse, respiratory rate, and temperature) were normal. On neurological examination, the patient’s pupils were isochoric and reacted to light promptly. Corneal and oculecephalic reflexes were intact. The patient showed responses to painful stimuli. Except for a blood glucose level of 10 mg/dl, all other blood laboratory tests (complete blood count, electrolytes, liver and renal functions, arterial blood gases) were normal. Intravenous dextrose was injected immediately, followed by glucose infusion and somatostatin treatment to restore the blood glucose level. As there was no doubt about the use of the oral hypoglycemic drug for suicidal purpose, a toxicology test was not performed.

**Figure 1:**
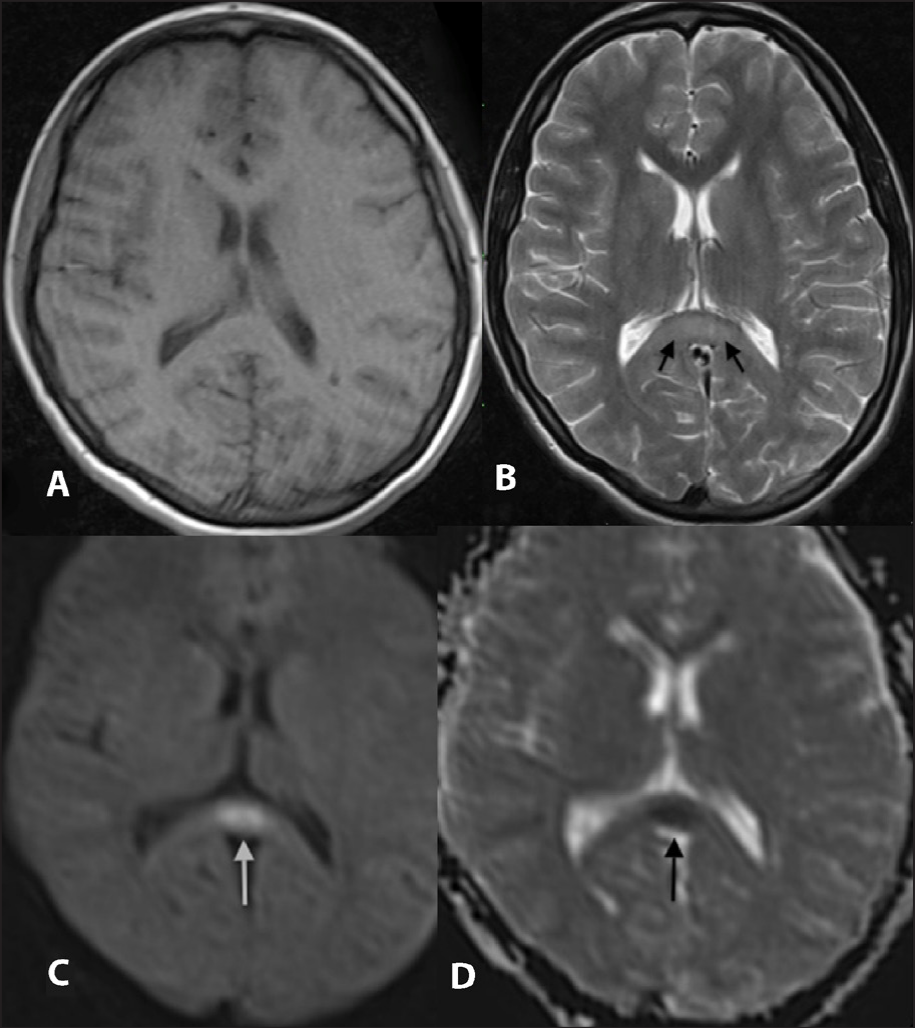
Results of imaging one hour after admission to the hospital. No lesion was observed on axial T1-weighted imaging (A), but axial T2-weighted imaging (B) showed a hyperintense lesion in the SCC (black arrows). First DWI (C) of profound hypoglycemia shows a high signal in the SCC (white arrow). An ADC map (D) at the same level shows diffusion restriction in the SCC (black arrow).

**Figure 2:**
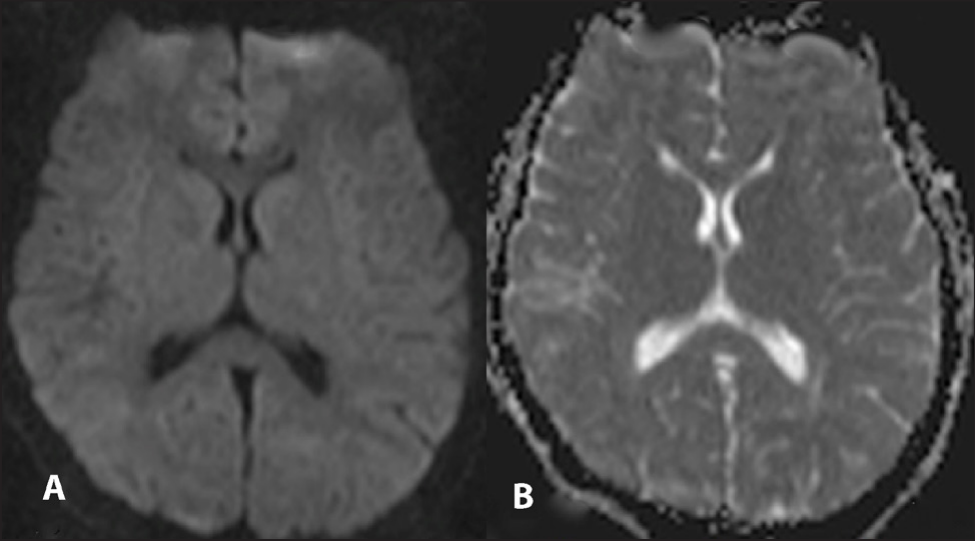
DWI (A) and ADC map (B) of the lesion two days later, showing complete resolution of the SCC lesion.

One hour after admission, a T2-weighted image on MRI (1.5 Tesla Siemens Magnetom Symphony Quantum, Erlangen, Germany) showed isolated focal hyperintensity in the SCC and a decrease in the apparent diffusion coefficient (ADC) compatible with diffusion restriction, in addition to a significant signal increase in the DWI (Fig. 1). After receiving treatment for 24 hours, the patient became conscious, and her blood glucose level returned to normal (90 mg/dl). DWI was repeated two days after the first MRI, and a control DWI (Fig. 2) showed complete resolution of the lesion in the SCC.

## Discussion

There are various causes of hypoglycemia, such as oral hypoglycemic drugs or insulin overdose, poor oral intake in diabetic and nondiabetics, undiagnosed insulinoma, sepsis, renal or hepatic failure, or Addison’s disease. Glucose is the brain main energy substrate. It has been shown in pathological studies that profound hypoglycemia may result in neuronal damage [1,2]. Hypoglycemia may also lead to a variety of neurological symptoms, such as transient motor deficits, deep memory loss, hemiparesis, irreversible coma, and even mortality in 2–4% of cases [2,7].

Studies describing MRI findings in hypoglycemic brain injury are limited, but those available have shown that typical lesions were in the cerebral cortex, basal ganglia, hippocampus, cerebral white matter, and internal capsule [2,4]. An isolated lesion of the SCC due to hypoglycemia as in the present case is very rare [3,5], but lesions of the SCC accompanied by corona radiata and/or an internal capsule have been reported more frequently [1,6,8,9].

Although neurological sequelae and the prognosis of hypoglycemic brain injury are associated with the duration and the severity of the hypoglycemia, it may be difficult to determine the duration in many cases [2,3]. According to some reports, initial blood glucose levels did not predict a poor outcome [3,4]. Although the initial blood glucose level was very low in the present case, only the SCC was involved, and the hypoglycemia showed complete resolution. Some studies showed that DWI may be useful in determining the prognosis in patients with hypoglycemic brain injury by localizing the lesions [1,2,3,4,8]. They also reported that the corpus callosum, internal capsule, and cerebral white matter lesions are usually reversible, without neurological deficit [1,2,6]. Cerebral cortex, basal ganglia, hippocampus, and white matter lesions were associated with poor outcomes in hypoglycemia, and DWI findings and the distribution of the lesions were useful in predicting the clinical outcomes [2,3].

Although the source of reversible diffusion restriction in SCC lesions in hypoglycemia is not clear, it may result from excitotoxic edema. Excitotoxic brain edema is a form of cytotoxic edema due to increased extracellular glutamate. Glutamate binding to NMDA (N-methyl-D-aspartate) receptors leads to calcium entry into the cell and apoptosis induction, whereas binding to non-NMDA receptors induces sodium entry and hence cytotoxic edema [9]. In contrast to cytotoxic edema, excitotoxic edema does not necessarily result in neuronal damage because glutamate-induced edema of glial cells and myelinic sheaths might protect axons from intracellular edema and irreversible damage. Additionally, glutamate reuptake systems are not impaired in hypoglycemia [2]. Thus, hypoglycemic brain injury in the SCC, internal capsule, and focal white matter is usually transitory, and DWI abnormalities normalize with time or following the elimination of the causative factors. Nevertheless, if glucose deprivation continues, energy failure, ionic pump failure, cellular calcium influx, and intracellular alkalosis may occur. Excitatory amino acid (aspartate) release into the extracellular space occurs and results in selective neuronal necrosis, predominantly in the cerebral cortex, caudoputamen, and hippocampus. Thus, diffusion restriction associated with hypoglycemia normalizes immediately after therapy but becomes persistent if treatment is delayed [2,6,9].

Hypoglycemia should be considered in reversible focal lesions of the SCC. Other common causes that present similar clinical findings are antiepileptic drug withdrawal or toxicity, viral encephalitis, Wernicke’s encephalopaty, alcohol use, high altitude cerebral edema, hemolytic uremic syndrome, electrolyte imbalance (hypo/hypernatremia), and Marchiafava–Bignami disease [5].

In conclusion, hypoglycemia, which can be confirmed with a simple blood test, should be kept in mind in comatose patients who are admitted to the emergency department. In hypoglycemic brain injury, lesion localization on DWI is vital to predict the prognosis.

## Competing Interests

The authors declare that they have no competing interests.
